# Gel formulated with *Bryophyllum pinnatum* leaf extract promotes skin wound healing *in vivo* by increasing VEGF expression: A novel potential active ingredient for pharmaceuticals

**DOI:** 10.3389/fphar.2022.1104705

**Published:** 2023-01-12

**Authors:** Edilane Rodrigues Dantas Araújo, Jacinthia Beatriz Xavier-Santos, Valéria Costa da Silva, Juliana Bessa Figueiredo de Lima, Jade Schlamb, Matheus de Freitas Fernandes-Pedrosa, Arnóbio Antônio da Silva Júnior, Raimundo Fernandes de Araújo Júnior, Thirumurugan Rathinasabapathy, Marvin Moncada, Debora Esposito, Gerlane Coelho Bernardo Guerra, Silvana Maria Zucolotto

**Affiliations:** ^1^ Postgraduate Program in Health Science, Federal University of Rio Grande do Norte (UFRN), Natal, Brazil; ^2^ Department of Pharmacy, Research Group on Bioactive Natural Products, Federal University of Rio Grande do Norte, Natal, Brazil; ^3^ Postgraduate Program in Pharmaceutical Science, Federal University of Rio Grande do Norte, Natal, Brazil; ^4^ Biotechnology and Technology Laboratory, Department of Pharmacy, Federal University of Rio Grande do Norte, Natal, Brazil; ^5^ Postgraduate Program in Drug Development and Technological Innovation, Federal University of Rio Grande do Norte, Natal, Brazil; ^6^ Plants for Human Health Institute, North Carolina State University, Kannapolis, NC, United States; ^7^ Cancer and Inflammation Research Laboratory, Morphology Department, Biosciences Center, Federal University of Rio Grande do Norte, Natal, Brazil; ^8^ Department of Food, Bioprocessing and Nutrition Sciences, North Carolina State University, Raleigh, NC, United States; ^9^ Department of Animal Science, NC State University, Raleigh, NC, United States; ^10^ Department of Biophysics and Pharmacology, Biosciences Center, Federal University of Rio Grande do Norte, Natal, Brazil

**Keywords:** Crassulaceae, topical gel formulation, wound healing, stability study, analytical marker, phenolic compounds

## Abstract

*Bryophyllum* *pinnatum* (Crassulaceae) is used in traditional medicine for treating skin wounds. In our previous study, a topical gel containing *B. pinnatum* aqueous leaf extract showed a preclinical anti-inflammatory effect in *in vivo* acute edema models. In continuation, the present study aims to evaluate the phytochemical content and the stability of a formulation in gel containing *B. pinnatum* aqueous leaf extract and its healing properties and mechanism of action through an experimental model of induction of skin wounds in rats and *in vitro* assays. The animals were treated topically for 7 or 14 days with a formulation in gel containing extract at 5% or a placebo or Fibrinase^®^ in cream. In addition, to establish some quality control parameters, the total phenolic content (TPC), total flavonoid content (TFC), and a study focusing on the phytochemical and biological stability of a gel for 30 days at two different conditions (room temperature and 40°C/75% RH) were performed. Gel formulation containing extract showed a TPC and TFC of 2.77 ± 0.06 mg of gallic acid/g and 1.58 ± 0.03 mg of quercetin/g, respectively. Regarding the stability study, the formulation in gel showed no significant change in the following parameters: pH, water activity, chromatographic profile, and the content of the major compound identified in the extract. The gel formulation containing extract stimulated skin wound healing while reducing the wound area, as well as decreasing the inflammatory infiltrate, reducing the levels of IL-1β and TNF-α, and stimulating angiogenesis with increased expression of VEGF, an effect similar to Fibrinase. In conclusion, the gel formulation containing extract exhibited relevant skin wound healing properties and, therefore, has the potential to be applied as a novel active ingredient for developing wound healing pharmaceuticals.

## 1 Introduction

The skin has many essential functions and is the largest organ of the integumentary system, with approximately 15% of body weight. Its most important function is being the primary barrier of the body, protecting the inner body structures from the environment (Takeo et al., 2015; Christophers and Schröder, 2021). In contrast, this attribute makes the skin the body region most exposed to damage, which could provoke serious injuries, such as ulcers and deep cuts. This damage can also be defined as skin wounds, which can be generated by physical, biological, and chemical factors (Varaprasad et al., 2020).

Wounds can be acute or chronic, and the wound repair process is categorized into four interconnected phases: 1) homeostasis/coagulation, 2) inflammatory cell recruitment, 3) proliferative phase, and 4) maturation phase (Martin and Nunan, 2015). In the first phase, platelets and the activation of the coagulation cascade are mainly involved. Fibrin strands adhere in this early stage, promoting the formation of a thrombus or a clot, trapping platelets in the wound area (Childs and Murthy, 2017). In the inflammatory phase, the recruitment of inflammatory cells occurs in the wound site to eliminate the damaged cells and pathogens in the wound area. First, neutrophils are recruited to the wound area and then monocytes, followed by other leukocytes. Swelling, heat, redness, and pain are the main signals generated by growth factors, enzymes, and chemokines produced by defense cells. However, when the inflammatory phase is longstanding and many activated cells are recruited out of control at the injured area, the inflammation will delay the wound healing process (Childs and Murthy, 2017; Dev et al., 2019).

In the sequence, the main function of the proliferative phase is to cover and fill the wound. In this phase, the wound edges begin contracting by fibroblasts that are activated and differentiated into myofibroblasts. After that, re-epithelialization starts, and this stage is caused by extracellular matrix deposition, mainly of collagen (Leavitt et al., 2016; Baron et al., 2020). Then, to finalize the healing process, during the maturation phase, collagen fibers are rearranged from collagen type III to type I, and the tissue is remodeled, gradually becoming firm and flexible by promoting epithelialization and neovascularization (Leavitt et al., 2016; Childs and Murthy, 2017; Baron et al., 2020).

Currently, synthetic wound healing agents are marketed, but with limitations that include high cost, emergence of multi-resistant organisms, and loss of efficacy. For this reason, research on new wound-healing pharmaceuticals containing botanical extracts has been gaining increasing interest (Yazarlu et al., 2021). Several studies have focused on the beneficial role of plant-derived products in wound management (Yaghoobi et al., 2013; Maver et al., 2015; Mssillou et al., 2022; Pan-Yue et al., 2022). In this scenario, our focus was on the plant *Bryophyllum pinnatum* (Lam.) Oken [synonymy: *Kalanchoe pinnata* (Lam.) Pers*.*] (Tropicos, 2022). This species belongs to the Crassulaceae family. It is popularly known as “saião” or “coirama” in Brazil, “planta da vida” in Mexico, and “canterbury” and “bells of cathedral” in the United States and Europe (Amaral et al., 2005; Herrera, 2009; Joseph et al., 2011; Fernandes et al., 2019). Crassulaceae has xeromorphic characteristics that allow its species to acclimatize to hot weather and insufficient hydration (Herrera, 2009).

*B. pinnatum* is native to Madagascar and has been well-adapted to Brazil, mainly to the Caatinga Biome (Allorge-Boiteau, 1996; Gehrig et al., 2001), but it is not an endemic species to Brazil (Zappi, 2015). Traditional medicine described the use of their leaves to treat skin wounds (Amaral et al., 2005). Several studies have reported the presence of phenolic compounds in their leaves in which flavonoid glycosides derived from quercetin, patuletin, eupafolin, and kaempferol are the compounds commonly described in the leaves of *B. pinnatum* (Muzitano et al., 2006; Afzal et al., 2012; Fernandes et al., 2016; De Araújo et al., 2018). The main compound reported in the literature for *B. pinnatum* is quercetin 3-*O*-α-L-arabinopyranosyl-(1→2)-*O*-α-L-rhamnopyranoside. It can be applied as an analytical marker in the quality control of raw material and products derived from *B. pinnatum* leaf extract (Dos Santos Nascimento et al., 2018; Fernandes et al., 2019; Coutinho et al., 2021; Morais Fernandes et al., 2021).

Due to the many ethnopharmacological reports attributed to *B. pinnatum*, several research groups have conducted studies to confirm their pharmacological or biological properties. In a review article published in 2019 (Fernandes et al., 2019), our research group gathered information about the traditional use of their leaves for treating the healing processes of skin and stomach wounds. A state-of-science review of this species revealed an extract with healing properties for treating ulcers (De Araújo et al., 2021), and a topical gel containing an extract showed potential to treat skin wounds (Coutinho et al., 2021); and another formulation in gel containing an extract had acute anti-inflammatory potential (De Araújo et al., 2019) in *in vivo* models. Specifically, this last study conducted by our research group encouraged us to follow a deep investigation to prove the wound healing efficacy of gel containing *B. pinnatum* leaf extract and understand its mechanism of action using the experimental model of induction of skin wounds in rats. In addition, stability studies of complex chemical mixture of herbal medicines present a key challenge compared to synthetic drugs (Klein-Junior et al., 2021), then this study also aimed to quantify the total phenolic content (TPC) and total flavonoid content (TFC) in a healing gel formulation and to determine its phytochemical and biological stability.

## 2 Materials and methods

### 2.1 Plant material

*Bryophyllum pinnatum* leaves were collected in cultivation in “Escola Agrícola de Jundiaí” at Universidade Federal do Rio Grande do Norte. The original samples were obtained from Horto Francisco José de Abreu at the Universidade Federal do Ceará (UFC) in Fortaleza city, Ceará State, Brazil, in October 2015. The botanical identification of the *B. pinnatum* species was performed by Dra. Rúbia Santos Fonseca, and a voucher specimen (n° 57335) was deposited at the Prisco Bezerra Herbarium of the Universidade Federal do Ceará, Brazil. The plant material collection was conducted under the authorization of the Brazilian Authorization and Biodiversity Information System (SISBIO) (process number 35017) and the research under the authorization of the National System for Management of Genetic Heritage (SISGEN) n° A7EA798. The legitimate scientific name of the species is *Bryophyllum pinnatum* (Lam.) Oken (synonymy: *Kalanchoe pinnata* (Lam.) Pers. (Tropicos, 2022).

### 2.2 Preparation of the *Bryophyllum pinnatum* leaf aqueous extract

Fresh *B. pinnatum* (3 kg) leaves were extensively washed and extracted with water by turbo extraction for 5 min in an industrial blender. A plant-to-solvent proportion of 1:1 (*w/v*) was used. The leaf aqueous extract was screened and concentrated by a rotary evaporator (model V-700, Buchi) at a temperature below 50°C. The concentrated aqueous extract was freeze-dried to obtain the dry extract. The aqueous *B. pinnatum* leaf extract yielded 231.0 g (4.40%).

### 2.3 Preparation of formulation in gel

The loaded *B. pinnatum* leaf extract in a gel formulation was performed as previously described in our study (De Araújo et al., 2019). The gel formulation was prepared with the following components: *B. pinnatum* leaf extract (5%, *w/w*), hydroxypropylmethylcellulose (HPMC) (2%, *w/w*), propylene glycol (PEG) (3%, *w/w*), poloxamer F-127 (15%, w/w), isopropyl alcohol (5%, *w/w*), methylparaben (0.2%, *w/w*), and distilled water q.s.p. Placebo gel was prepared with the same components, except for the *B. pinnatum* leaf extract. Phase A: Polaxamar F-127, *B. pinnatum* extract, and isopropyl alcohol were solubilized slowly in distilled cold water with continuous stirring in a homogenizer and finally added to phase B, which comprises HPMC, PEG, and methylparaben with stirring until complete homogeneity. Finally, a green-colored gel was obtained. It was stored in a light-resistant airtight package at 4°C for 24 h (considered day 0 of formulation in gel). The batch was divided into three parts: a wound healing *in vivo* model, phenol and flavonoid content assays, and a stability study.

### 2.4 Phytochemical study

#### 2.4.1 Ultra-high-performance liquid chromatography profile of the *Bryophyllum pinnatum* leaf extract

The ultra-high-performance liquid chromatography coupled to a mass spectrometer. The column used was a Shimadzu Shim-pack XR-ODS (50 × 3.0 mm × 2.2 μm). The eluents were (A) 0.1% formic acid and (B) 0.1% formic acid in acetonitrile for positive mode and (A) water and (B) acetonitrile for negative mode. The gradients used were 0–30 min 10%–30% B, 30–31 min 30%–100% B, 31–35 min 100% B, 35–36 min 100%–10% B, and 36–40 min 10% B. The mobile phase temperature was set at 30°C, and the injection volume was 20 μL. For mass spectrometry, the capillary was adjusted to 4 KV, and nitrogen was used as a nebulizer and dryer gas at 300°C, 8 L/min, and 35 psig. The acquisition was performed as a scan mode in the negative mode for *m/z* 100–1,700 and in the positive mode for *m/z* 100–2,000.

#### 2.4.2 Determination of total phenolic and flavonoid content of a formulation in gel containing *Bryophyllum pinnatum* leaf extract at 5%

The formulation in gel containing *B. pinnatum* leaf extract at 5% and placebo was prepared, as shown in Section 2.3. A total of 3 g of gel was divided into three beakers (1 g/each), extracted with 10 ml of methanol, and then stirred for 10 min (DiagTech^®^ magnetic stirrer, model DT3110H). After that, each sample was transferred to falcon tubes and centrifuge (Eppendorf^®^ Centrifuge, model 5804/5804R) at 24°C, 4,500 rpm, for 10 min. The supernatant was collected using a pipette and concentrated in a rotavapor (Buchi^®^, model R-210) at a temperature below 50°C. The decanted product (gel) was again extracted with 10 ml of methanol, and the entire process was repeated until the flavonoid extraction was complete. To ensure all flavonoids were extracted from the gel, the extraction process was carried out through thin layer chromatography (TLC) (described below). The standard quercetin 3-*O*-*α*-L-arabinopyranosyl-(1→2)-*O*-*α*-L-rhamnopyranoside, named Bp1, the main compound identified in *B. pinnatum* leaf extract, was used as a control. Bp1 standard was obtained and identified in our previous publication (Morais Fernandes et al., 2021). Thus, the extraction process of a formulation in gel was repeated five times until the entire content of flavonoids from the gel was extracted. Five solution extractions were gathered and dried at rotavapor at a temperature below 50°C to determine the TPC and TFC, respectively, per gram of formulation in gel through a spectrophotometer. The same procedure was repeated for the placebo gel formulation.

#### 2.4.3 Analysis by thin layer chromatography

TLC was a tool used to check the number of extractions required for the entire flavonoid extraction from gel. Each solution (1–5) contains flavonoids extracted from gel; the solution obtained from the extraction of placebo formulation, the solution of a free leaf extract from *B. pinnatum*, and the standard Bp1 were solubilized in methanol and then analyzed by TLC using silica gel 60 F254 aluminum plates (Macherey-Nagel, Düren, Germany) as an adsorbent. As the mobile phase, the following system was used: ethyl acetate: formic acid: methanol: water (10:0.5:0.6:0.2, *v/v/v/v*). Chromatoplates were sprayed with 0.5% Natural Product Reagent A (diphenylboric acid *β*-aminoethyl ester), a specific reagent to detect flavonoids, and visualized under UV at 365 nm.

#### 2.4.4 Determination of TPC in formulation in gel containing *Bryophyllum pinnatum* leaf extract

The quantitative determination of TPC in the formulation in gel was performed using a UV–visible spectrophotometer through the Folin–Ciocalteu method (Singleton and Rossi, 1965) with few adaptations. The solution obtained from extractions of formulation in gel containing *B. pinnatum* leaf extract was prepared at concentrations of 4 and 2 mg/ml. The same procedure was repeated with the solution obtained from the extraction of the placebo gel. The standard gallic acid at a concentration of 2.5–100 μg/ml was used. A volume of 25 μL of was added in triplicate in a 96-well microplate. Then, 125 μL of an aqueous solution of Folin–Ciocalteu reagent at 10% (*v/v*) and 120 μL of sodium bicarbonate at 7.5% (*w/v*) were added. The mixture was incubated for 30 min, protected from light (covered with aluminum foil), at room temperature. The absorbance (λ = 760 nm) was measured with a microplate spectrophotometer (Mindray^®^ microplate reader, model MR-96A). The TPC was quantified by means of interpolation on a standard curve prepared with gallic acid and expressed as milligrams of gallic acid per gram of dry extract and per gram of the gel formulation. The analysis was performed intra-day (in triplicate) and inter-day (three different days).

#### 2.4.5 Determination of TFC in formulation in gel containing *Bryophyllum pinnatum* leaf extract

The quantitative determination of TFC in the formulation in gel was performed using a UV–visible spectrophotometer through a method described in the German Pharmacopoeia (Knabe, 1979) and adapted by Woisky and Salatino (1998). The solution obtained from extractions of formulation in gel containing *B. pinnatum* leaf extract was prepared at concentrations of 4 and 2 mg/ml. The same procedure was repeated with a solution obtained from extractions of placebo gel. The standard quercetin at a concentration of 5 to 200 μg/ml was used. Aliquots (50 μL) of samples were added in triplicate in a 96-well microplate. Subsequently, 160 μL of ethanol P.A., 20 μL of 1.8% (*w/v*) aluminum chloride aqueous solution, and 20 μL of sodium acetate aqueous solution (820.3 mg in 100 ml) were added. The mixture was incubated for 40 min, protected from light (covered with aluminum foil), and kept at room temperature (24°C–28°C). The absorbance (λ = 415 nm) was measured with a microplate spectrophotometer (Mindray^®^ microplate reader, model MR-96A). The TFC was quantified through the interpolation of a standard curve of quercetin and expressed as milligrams of quercetin per gram of dry extract and per gram of gel formulation. The analysis was performed intra-day (in triplicate) and inter-day (three different days).

### 2.5 Stability study of formulation in gel

The stability study was conducted by formulation in gel for 30 days at two different conditions: 22°C–25°C (room temperature) and in a climate chamber (PHCbi) at 40°C/75% relative humidity (RH). For each endpoint (0, 15, and 30 days) and condition (22°C–25°C and 40°C/75% RH), three packages containing gel were prepared (4 g per package). The following analyses were performed in triplicate: pH, water activity, chromatographic profile, and quantification of Bp1 content through HPLC-DAD.

#### 2.5.1 pH

A 10% (*w/v*) dilution of each formulation in gel in water was prepared, and pH was checked using Orion Star A 211 pH meter (Thermo Scientific^®^). The gels were analyzed after 0, 15, and 30 days (*n* = 3 packages at room temperature and *n* = 3 packages at 40°C/75% RH) in triplicate.

#### 2.5.2 Water activity

Water activity (wa) was determined by sample direct-reading, using the Aqualab equipment, model 3 TE (Decagon Devices, Pullman, WA, USA), at a temperature of 25°C. A sample of formulation in gel (*n* = 3 packages at room temperature and *n* = 3 packages at 40°C/75% RH) was analyzed in triplicate.

#### 2.5.3 Phytochemical stability through HPLC-DAD

The phytochemical stability of formulation in gel containing *B. pinnatum* leaf extract was performed by HPLC-DAD to compare the chromatographic profile and the content of Bp1 of the leaf extract for 30 days. After the endpoints (0, 15, and 30 days), an aliquot of formulation in gel (1 g of each package, *n* = 3 for packages at room temperature, and *n* = 3 for packages at 40°C/75% RU) was extracted with 1 ml of methanol, with stirring for 40 min. Subsequently, the content was transferred to falcon tubes and centrifuged (4,000 rpm, 5 min). The supernatant was collected and dried in the rotavapor (R-300 Buchi Switzerland^®^) at a temperature below 50°C. Solutions of 50 mg/ml (methanol: water, 1:1, *v/v*) were prepared and analyzed in triplicate, and the relative standard deviation was calculated according to the peak area of Bp1 flavonoid (major compound identified at *B. pinnatum* leaf extract). Bp1 standard was solubilized in 1:1, methanol: water (*v/v*). Analysis was undertaken using a Shimadzu Prominence LC system equipped with a Degasser (DGU-20A 5R), Pump (LC 20 AR), Autosampler (SIL-20A HT), Column Oven (CTO-20A), and Diode Array Detector (SPD-M20A). A Phenomenex Luna C18 guard column (10 × 2.1 mm, 5 μm) and a Phenomenex Luna C18 column (250 × 4.6 mm × 5 μm) were used at a solvent flow rate of 1 ml min^−1^. The mobile phase comprised A (0.1% formic acid in water) and B (acetonitrile). The following gradient (*v/v*) was applied: 10% B, 0–5 min, 10%–55% B, 5–35 min, 55%–95% B, 35–37 min, 37–39 min, 95% B, and a subsequent re-equilibration period 10% B, 39–45 min. The temperature of the mobile phase was set at 30°C, and the injection volume was 20 μL. The DAD was set at 261, 274, 310, and 324 nm to record the peak intensities. The chromatographic evaluation was conducted using Shimadzu LabSolutions software. The Bp1 peak was confirmed by the retention time, UV spectrum, and co-injection of Bp1 standard + extract through observation of the increased peak area.

#### 2.5.4 Biological stability through *in vitro* reactive oxygen species assay

The biological stability of formulation in gel containing *B. pinnatum* leaf extract through reactive oxygen species (ROS) *in vitro* analysis was assayed using murine macrophage (RAW 264.7) cells obtained from American Type Culture Collection (ATCC, Livingstone, MT, USA) (ATCC® TIB-71TM). Cells were routinely maintained in Dulbecco’s modified Eagle’s medium (DMEM, Life Technologies, Grand Island, NY, USA), supplemented with 100 μg/ml penicillin and 100 μg/ml streptomycin (Penstrep, Gibco, Life Technologies, REF#15140-122), and 10% (*V/V*) fetal bovine serum (FBS, Life Technologies, Long Island, NY, ISA). Approximately 2.8 × 10^5^ cells/ml were kept at 37°C and 5% CO_2_ in a humidified incubator. The cells were seeded into a sterile 24-well plate (NunclonTM Delta Surface, Thermo Scientific) with DMEM. After adhesion and confluence over 24 h, cells were exposed to a fresh fluorescent medium of 50 µM solutions of dichlorodihydrofluorescein diacetate acetyl ester (H_2_DCFDA) in ethanol for 30 min. The medium was aspirated, and the cells were treated with 1 µL of the *B. pinnatum* free leaf extract or with solutions extracted from gel containing *B. pinnatum* leaf extract or the negative (80% ethanol) and positive controls of 10 µM ammonium pyrrolidinedithiocarbamate (PDTC) or 10 µL of lipopolysaccharide (LPS, from *Escherichia coli* 127: B8, 100 μg/ml). Then, the cultured cells were incubated for 24 h. The fluorescence of 20,70-dichlorofluorescein (DCF) was measured at 485 nm (excitation) and 515 nm (emission) on a microplate reader (Synergy H1, Biotech, Winooski, VT, USA) using the Gen 5TM software program (Take 3 Session, Biotek, Winooski, VT, USA). The results were expressed as ROS production (%) relative to LPS induction. Each sample was analyzed in triplicate.

### 2.6 *In vivo* study

Female Wistar rats (180–250 g) 8–10 weeks old were obtained from the Vivarium Health Center of the Health Sciences Center at Federal University do Rio Grande do Norte (UFRN). They were kept under standard environmental conditions (12-h dark/light cycle) and temperature (22°C ± 2°C). Water and industrialized dry food (Presence, Purina^®^, Brazil) were made available *ad libitum*. All the experiments were conducted in accordance with the National Council for the Control of Animal Experimentation of Brazil (CONCEA), the International Guiding Principles for Biomedical Research Involving Animals of the Council of International Organizations of Medical Sciences (CIOMS), and were submitted to and approved by the Ethics Committee on Animal Use at Universidade Federal do Rio Grande do Norte (UFRN), under license no. 029.047–2017. The anesthesia was performed with xylazine (10 mg/kg, i.p.) and ketamine (80 mg/kg, i.p.). The animals were euthanized with an overdose of xylazine (30 mg/kg, i.p.) and ketamine (240 mg/kg, i.p.).

#### 2.6.1 Wound healing activity

The assay occurred following Kim et al. (2017), with some adaptations described below. The animals were randomly divided into three groups (*n* = 10/group) and treated daily topically. Group 1 (placebo control) was treated with 50 mg of placebo (base gel, without incorporation of *B. pinnatum* leaf extract), group 2 was treated with 50 mg of Fibrinase^®^ in cream (fibrinolysin 1 U/g, deoxyribonuclease 666 U, and chloramphenicol 0.01 g/g), and group 3 was treated with 50 mg of the gel formulation containing 5% of *B. pinnatum* leaf extract. All animals were kept in individual cages until the end of the experiment. Before inducing the wounds, the animals were submitted to intraperitoneal anesthesia with ketamine and xylazine (80 and 10 mg/kg, respectively) and placed in the prone position for back shaving. After asepsis with 70% alcohol, the excisional skin wounds were made in duplicate by pressing the skin of the dorsal region of each animal with a circular biopsy punch of 6 mm in diameter followed by a scissor cut to obtain an area of 12 mm.

Topical treatment started immediately after induction and was then performed every day for 14 days, once a day and always at the same time. The injured area was photographed, and its dimension was measured using the ImageJ software (National Institute of Health, Bethesda, MD) on the 1st, 7th, and 14th days of treatment. Results were expressed as wound area closure in mm^2^. At the end of each period (7th and 14th day), five animals from each group were euthanized with an overdose of ketamine and xylazine, and a wound biopsy was performed for further analysis of cytokine levels, histological analysis (H&E), and immunohistochemistry (VEGF).

#### 2.6.2 IL-1β and TNF-α assay

The wound skin samples were homogenized and processed as described by Safieh-Garabedian et al. (1995). The levels of interleukin-1*β* (IL-1β) [detection range: 62.5–4,000 pg/ml; sensitivity or lower limit of detection (LLD): 12.5 ng/ml of recombinant mouse IL 1*β*] and tumor necrosis factor-*α* (TNF-α) (detection range: 62.5–4,000 pg/ml; sensitivity or LLD: 50 ng/ml of recombinant mouse TNF-α) in the wound skin samples were determined with a commercial ELISA kit (R&D Systems, Minneapolis, MN, United States), as previously described. All samples were within the wavelength used in UV–VIS spectrophotometry (absorbance measured at 490 nm).

#### 2.6.3 Histological analysis

The skin wound biopsy specimens were fixed in 10% buffered formalin, dehydrated, and paraffin-embedded. Then, 4-µm-thick samples were obtained for hematoxylin–eosin (H&E) staining and examined by light microscopy (Nikon E200 LED, Minato, Tokyo, Japan). Three sections of the lesions (five animals per group) were analyzed. Morphological changes were investigated using scores whose parameters (Abramov et al., 2007) are shown in Table 1. Reported histopathological analyses were independently performed by two pathologists blinded to the group identity. For the readings, Planimetry microscopy (Nikon E200 LED, LAICI, Morphology Department/UFRN) with an objective lens at ×10 and ×40 magnification was used.

**TABLE 1:** Criteria for histological analysis of healing.

Scores	Inflammatory infiltrate	Neovascularization	Re-epithelization	Granulation	Crust and necrosis
0	Absent	Absent	Absent	Absent	Absent
1	Discrete	Initial	Partial	Present	Present
2	Moderate	Partial	Complete	—	—
3	Intense	Complete	—	—	—

#### 2.6.4 Immunochemistry

Thin wound skin biopsy sections (3 µm) were obtained from each group with a microtome and transferred to gelatin-coated slides. Each tissue section was then deparaffinized and rehydrated. The wound biopsy slices were washed with 0.3% Triton X-100 in phosphate buffer (PB) and quenched with endogenous peroxidase (3% hydrogen peroxide). Tissue sections were incubated overnight at 4°C with primary antibodies (Santa Cruz Biotechnology, Interprise, Santa Cruz, CA, USA) against VEGF and primary antibody (Spring-Abcam, Massachusetts, USA). Dilution tests (three dilutions) were performed with all antibodies to identify the 1:100 dilution as appropriate. Slices were washed with a phosphate buffer and incubated with a streptavidin/HRP-conjugated secondary antibody (Biocare Medical, Concord, CA, USA) for 30 min. Immunoreactivity to the various proteins was visualized with a colorimetric-based detection kit following the protocol provided by the manufacturer (TrekAvidin-HRP Label + Kit from Biocare Medical, Dako, CA, USA). Sections were counter-stained with hematoxylin. Known positive controls and negative controls were included in each sample set. Planimetry microscopy (Nikon E200 LED, Morphology Department/UFRN) with a high-power objective (×10 and ×40) was utilized to score the intensity of cell immunostaining, according to the methodology used by Araújo Júnior et al. (2014).

### 2.7 Statistical analysis

All values were reported as the mean ± standard mean error or as mean ± standard deviation and were analyzed by one-way or two-way ANOVA followed by Tukey or Bonferroni *post hoc* test for multiple comparisons. Non-parametric data (score) are expressed as the median (range) and were analyzed using the Mann–Whitney test. All statistical analyses were performed using GraphPad 8.0 software (GraphPad Software Inc., La Jolla, California, USA), and statistical significance was set at *p* < .05.

## 3 Results

### 3.1 UHPLC-MS profile of the *Bryophyllum pinnatum* leaf extract

The chromatographic profiles through UHPLC-MS allowed the characterization of peaks with similar mass spectra to flavonoid glycosides, with a nucleus of quercetin, patuletin, eupafolin, and kaempferol in *B. pinnatum* leaf extract (Table 2). The characterization of *B. pinnatum* leaf extract was shown in detail by De Araújo et al. (2018).

**TABLE 2:** Compounds characterized through UHPLC-MS in *B. pinnatum* free leaf extract.

Peak	Retention time	[M + H]^+^	[M − H]^−^	Compounds
1	3.41	641.1353		Patuletin-*O*-deoxy-hexoside-*O*-hexoside
2	4.88		463.0937	Quercetin-*O*-hexoside
3	9.86	581.1527	579.1425	Quercetin-*O*-deoxy-hexoside-*O*-pentoside
4	10.08		579.1426	Quercetin-*O*-deoxy-hexoside-*O*-pentoside
5	10.51	581.1513	579.1421	Quercetin-*O*-deoxy-hexoside-*O*-pentoside
6	13.03	565.1558		Kaempferol-*O*-deoxy-hexoside-*O*-pentoside
7	13.81	595.1667		Eupafolin-*O*-deoxy-hexoside-*O*-pentoside

### 3.2 Determination of the TPC and TFC content in formulation in gel containing *Bryophyllum pinnatum* leaves

**FIGURE 1:**
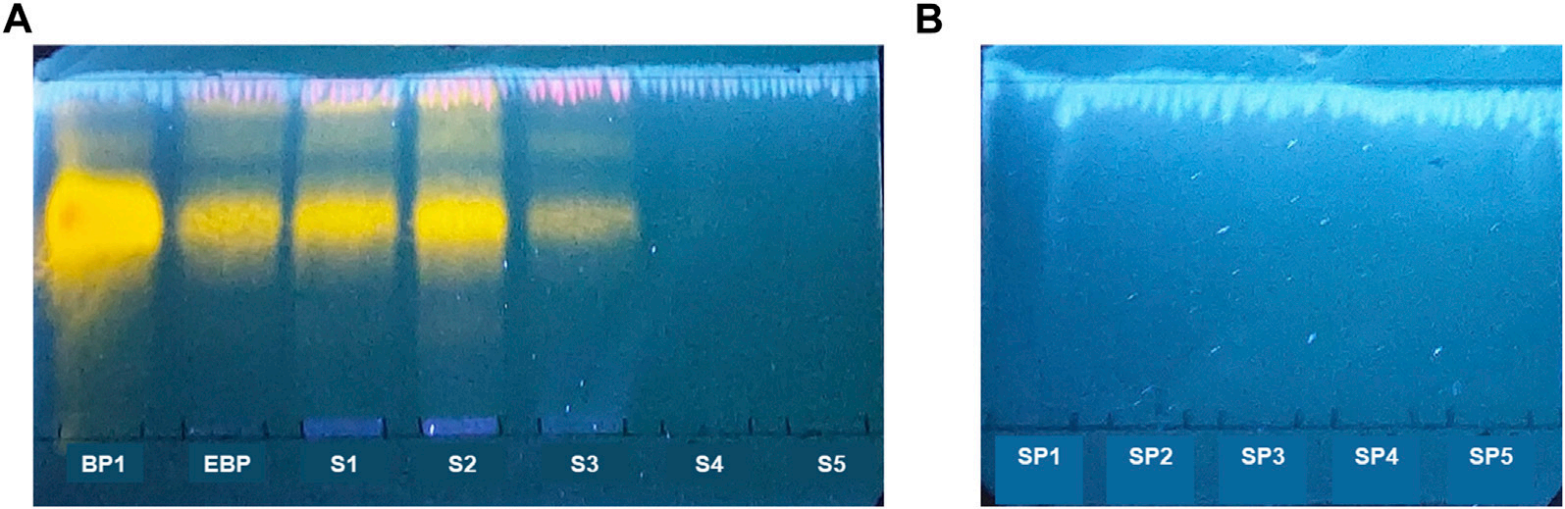
TLC of the gel formulation containing *B. pinnatum* leaf extract. Mobile phases: ethyl acetate: formic acid: methanol: water (10:0.5:0.6:0.2, *v/v*/*v/v*). Adsorbent: silica gel 60 F254. Detection: Natural Product Reagent A at 0.5%. Visualization: UV 365 nm. **(A)** Solution extractions with methanol from the gel containing *B. pinnatum* leaf extract (S 1–5). Bp1, standard quercetin 3-*O*-*α*-L-arabinopyranosyl-(1→2)-*O*-*α*-L-rhamnopyranoside; EBP, *B. pinnatum* leaf extract. **(B)** Solution extractions of the placebo gel (SP = 1–5).

The solutions extracted from gel containing *B. pinnatum* leaf extract and from placebo gel were analyzed by TLC. Then, plates were sprayed using Natural Product Reagent A and observed under UV light at 365 nm to possibly visualize yellow zones (Figure 1A **)**, characteristic of the presence of flavonoids (Wagner and Bladt, 2001). Figure 1A shows the presence of yellow zones in the *B. pinnatum* free leaf extract (second column) and in the solutions obtained from the extraction from gel formulation containing *B. pinnatum* leaf extract (third, fourth, and fifth columns). In the first column (Figure 1A **)**, the strong yellow color (*Rf* = 0.62) represents the standard Bp1, named quercetin 3-*O*-*α*-L-arabinopyranosyl-(1→2)-*O*-*α*-L-rhamnopyranoside. The same yellow color zone with the same *Rf* was observed in the *B. pinnatum* leaf extract and in the solution extractions obtained from the gel formulation containing extract. Figure 1B shows the TLC of the solution extractions obtained from the placebo gel. Figure 1B does not show the yellow zone associated with the presence of flavonoids after spraying the Natural Product Reagent A. It was observed that at least three methanol extractions of gel were required to ensure the entire extraction of flavonoids from formulation in gel. In the sequence, solutions 1–5 were pooled, and TPC and TFC in gram per gel were determined.

The TPC of the gel formulation containing *B. pinnatum* leaf extract at 5% was determined according to calibration curves on three different days of analysis, using gallic acid as a standard at concentrations from 2.5 to 100 μg/ml and with Pearson’s correlation coefficient ® greater than 0.99 (Table 3). The TPC was expressed in gallic acid equivalent (mg) per gram of weight of the gel formulation and is shown in Table 3, with a mean value equal to 2.77 ± 0.06 mg in 1 g of the gel formulation. The TFC in the gel formulation containing extract was calculated using the equation of the straight line derived from the calibration curve of the quercetin standard (5–200 μg/ml), obtaining *r* > 0.99 (Table 3). The data were expressed in quercetin equivalent (mg) per gram of weight of the gel formulation and shown in Table 4, with a mean value equal to 1.58 ± 0.03 mg in 1 g of the gel formulation. Our results suggest that a formulation in gel containing *B. pinnatum* leaf extract at 5% has a TPC of 2.77 ± 0.06 mg and TFC content of 1.58 ± 0.03 mg per gram of gel, and this content ensures the wound healing properties. It is considered an essential parameter in the quality control of herbal products to guarantee the reproducibility of the pharmacological effect.

**TABLE 3:** Quantification of TPC and TFC in the gel formulation containing the *B. pinnatum* leaf extract at 5%.

Standard	Days	Gel 1	Gel 2	Gel 3	Mean	Placebo gel	*r*
TPC (milligram of gallic acid/g in 1 g of the gel formulation)	Day 1	2.41 ± 0.07	3.02 ± 0.05	2.19 ± 0.06	2.54 ± 0.06	ND	0.9957
	Day 2	2.33 ± 0.04	3.01 ± 0.05	2.98 ± 0.06	2.77 ± 0.05		0.9991
	Day 3	2.78 ± 0.08	3.07 ± 0.07	3.19 ± 0.09	3.01 ± 0.08		0.9978
Mean					2.77 ± 0.06		0.9975
TFC (milligram of quercetin/g in 1 g of gel formulation)	Day 1	1.16 ± 0.02	1.99 ± 0.01	0.99 ± 0.01	1.38 ± 0.01	ND	0.9995
	Day 2	1.33 ± 0.02	2.01 ± 0.04	1.75 ± 0.01	1.70 ± 0.03		0.9985
	Day 3	1.62 ± 0.01	1.79 ± 0.04	1.58 ± 0.03	1.66 ± 0.03		0.9997
Mean					1.58 ± 0.03		0.9986

Gel 1–3, formulation in gel containing *Bryophyllum pinnatum* leaf extract at 5%. ND, not detected. Data represented as mean ± standard deviation (*n* = 3). *r*, Pearson’s correlation coefficient.

**TABLE 4:** Stability study of formulation in gel containing *B. pinnatum* leaf extract at 5%.

	pH							Water activity						
	22°C–25°C*				40°C/75% RH**			22°C–25°C*				40°C/75% RH**		
Gel	0 day***	15 days	30 days	RSD%	15 days	30 days	RSD%	0 day***	15 days	30 days	RSD%	15 days	30 days	RSD%
1	4.50	4.40	4.80	3.84	4.24	4.20	3.17	0.9998	0.9786	0.9844	1.10	0.9775	0.9758	1.36
2	4.48	4.34	4.47	1.76	4.32	4.26	2.61	0.9985	0.9802	0.9760	1.21	0.9764	0.9820	1.16
3	4.46	4.30	4.50	2.39	4.43	4.30	1.93	0.9733	0.9802	0.9614	0.99	0.9864	0.9788	0.67

Phytochemical stability-Peak area of Bp1-HPLC-DAD (μg/50 mg of soluction extracted from gel)							
	22°C–25°C*				40°C/75% RH**		
Gel	0 day***	15 days	30 days	RSD%	15 days	30 days	RSD%
1	272.6	260.0	265.9	1.8	281.0	229.5	10.6
2	277.0	254.9	261.5	4.26	264.0	254.5	6.1
3	275.0	254.8	275.8	4.39	242.9	254.5	5.1

*Room temperature (on the bench). **RH, relative humidity; ***0 day, 24 h after gel preparation and stored at 4°C. Data are presented as a means of triplicate analysis. RSD of 40°C/75% RH was calculated using 0 (22°C–25°C), 15, and 30 days (40°C/75% RH).

### 3.3 Stability study of formulation in gel

**FIGURE 2:**
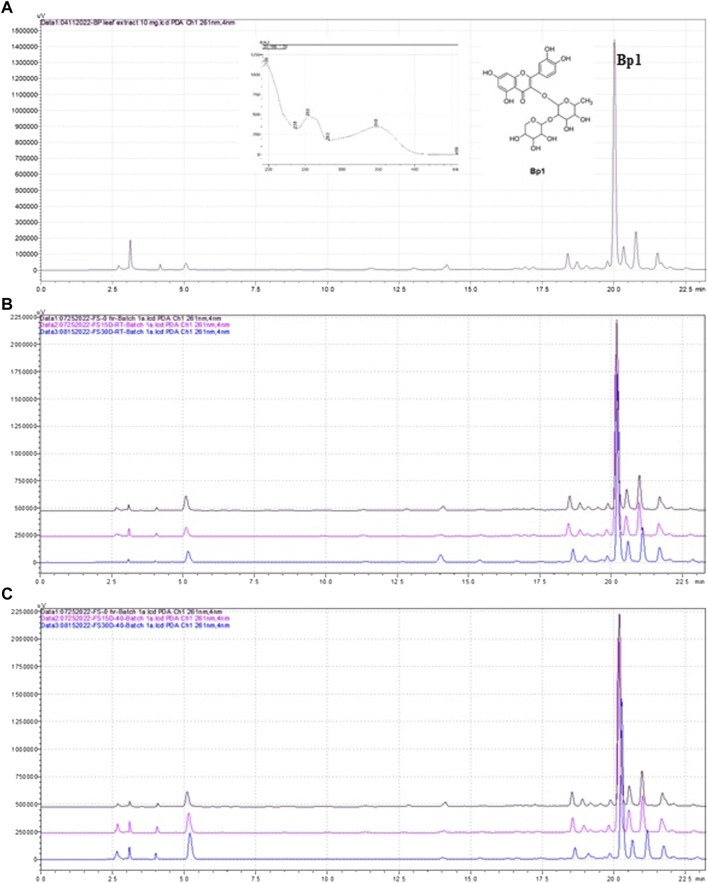
Chromatographic profile obtained by HPLC-DAD of *B. pinnatum* leaf extract **(A)**, solution extracted from gel 1 at room temperature (22°C–25°C) at different endpoints (0, black line, 15 days, pink line, and 30 days, blue line) **(B)** and the solution extracted from gel 1 at 40°C/75% HR at different endpoints (0, black line, 15 days, pink line, and 30 days, blue line) **(C)** Stationary phase: Phenomenex Luna C18 (150 × 4.6 mm, 2.6 mm) equipped with a Phenomenex Luna C18 guard column (10 mm × 2.1 mm, 5 μm). Mobile phase: A (0.1% formic acid in water) and B (acetonitrile); 10% of B for 5 min, gradient until 55% B at 35 min, to 95% B at 37 min, maintained for 2 more min at 95%, and a subsequent re-equilibration period of 6 min 10% B; flow elution 1 ml/min; detection 261 nm.

The stability of gel was studied in two conditions: at room temperature (22°C–25°C) and at 40°C/75% RU. The formulation was checked after 0 (24 h of preparation), 15, and 30 days for the change in pH, water activity and chromatographic profile, and content of Bp1. Similar chromatographic profiles were observed in all batches at room temperature and at 40°C/75% **(** Figure 2). Table 3 shows the observations of the formulation in gel during the storage period of 30 days. Regarding the pH, it was observed that the mean was 4.48–4.59 for 0–30 days at room temperature and 4.31–4.39 at 40°C/75% RU (Table 4). Thus, no significant changes were observed in pH formulation in gel. Concerning the water activity (Table 4) of formulation in gel, although it showed a high value, no growth of bacteria, yeast, or mold was observed, and no significant changes were observed during 30 days of products kept on the bench and at accelerated conditions of temperature and relative humidity. In addition, during 30 days of the stability study and the determination of the chromatographic profile and content of the major flavonoid of *B. pinnatum* leaf extract, Bp1 was used in the evaluation of phytochemical stability through HPLC analysis. Bp1 showed a retention time (*Rt*) of 20 min at *B. pinnatum* leaf extract, and the UV spectra showed two major absorptions maxima, 256 and 283 nm II band, and 348 nm I band (Figure 2). A similar chromatographic profile was observed for solutions extracted from gels at room temperature and at 40°C/75% RH. Bp1 was easily detected (*Rt* = 20.2 min) in all gel samples in the different endpoints and conditions. Table 3 shows the Bp1 content in formulation in gel kept on the bench at room temperature and at 40°C/75% RH. No significant change in the content of Bp1 was observed in the product kept at room temperature, and an RSD higher than 5% was found in products kept at 40°C/75% RH, but the chromatographic profile of the entire extract remained similar to free extract (Figure 2A). Figure 2 shows an illustrative chromatographic profile of free extract **(** Figure 2A) and solution extract from gel 1 at room temperature (22°C–25°C) **(** Figure 2B **)** and at 40°C/75% RH (Figure 2C). The formulation in gel was stable at the conditions studied for 30 days.

**FIGURE 3:**
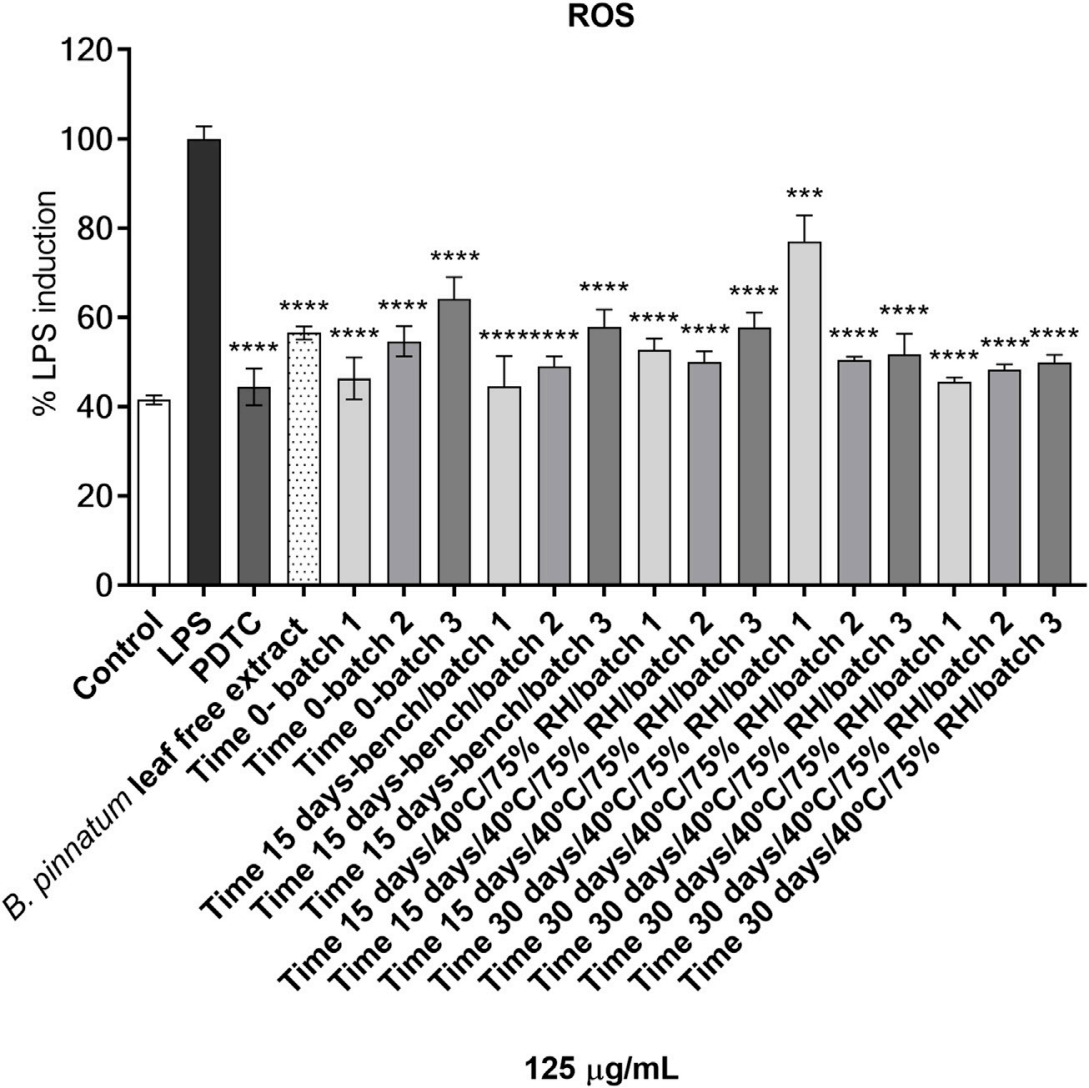
The effect on intracellular reactive radical species (ROS) of the *B. pinnatum* leaf free extract and the solutions extracted from gel containing *B. pinnatum* leaf extract at 125 μg/ml on murine macrophage RAW 264.7 cells. ROS production, control (80% ethanol), LPS (100 µg/lipopolysaccharide), PDTC (10 μM, pyrrolidinedithiocarbamate ammonium); one-way ANOVA was followed by the *post hoc* Dunnett test. ***p < 0.001, ****p < 0.0001 *vs*. LPS-treated cells. Bench = room temperature (22–25°C).

Figure 3 shows that the *B. pinnatum* free leaf extract and the solutions extracted from gel containing *B. pinnatum* leaf extract for 30 days at room temperature and 40°C/75% RU at 125 μg/ml suppressed the generation of ROS in LPS-treated murine macrophage RAW 264.7 cells compared with untreated LPS cells. ROS in LPS-treated RAW 264.7 cells was chosen to evaluate the biological stability because it is a viable method to evaluate a large number of samples, and in skin lesions, inflammatory cells synthesize free radicals and ROS to the defense against pathogens and mediate important intracellular pathways for the resolution of the inflammatory phase (Newbern, 2018; Gonçalves et al., 2022). However, the excessive production of free radicals and ROS promote tissue oxidative stress, causing deleterious effects on cell membranes, proteins, and nucleic acids, consequently prolonging the healing time of the wound, which may facilitate the development of infections in the affected region (Newbern, 2018). In this sense, our results proved the biological stability of formulation in gel containing *B. pinnatum* leaf extract for 30 days.

### 3.4 *In vivo* study

#### 3.4.1 Macroscopic analysis of the skin wound area

**FIGURE 4:**
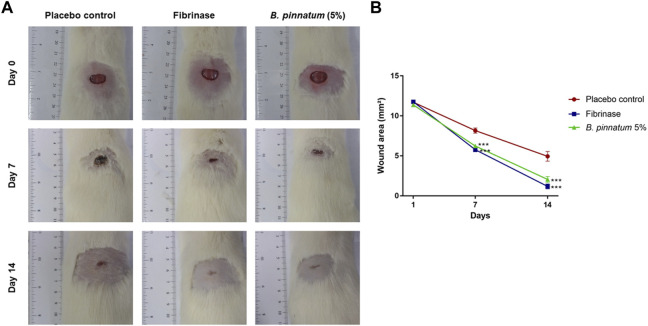
**(A)** Effect of topical treatment with a gel formulation containing *B. pinnatum* leaf extract at 5% on the skin wound area in rats. **(B)** Results expressed as mean ± standard error of mean (*n* = 5/group). ***p < 0.001 *vs*. placebo control by two-way ANOVA (post-Bonferroni test).

Figure 4 shows the healing process of skin wounds for 14 days. On the first day of the experiment (day 0), wounds were induced on the back of all animals with an area of 12 mm in diameter. Topical treatment with gel formulation containing *B. pinnatum* leaf extract was able to accelerate the wound healing process, significantly reducing the wound area on the 7th (*p* < 0.001) and 14th (*p* < 0.001) days compared with the control group treated with placebo gel (Figure 4B). Topical treatment with Fibrinase^®^ (positive control), as expected, was also able to accelerate the healing process, significantly reducing the wound area on the 7th (*p* < 0.001) and 14th (*p* < 0.001) days compared with the placebo control group (Figure 4B).

#### 3.4.2 Effect of gel formulation containing *Bryophyllum pinnatum* leaf extract at 5% on IL-1β and TNF-α levels

**FIGURE 5:**
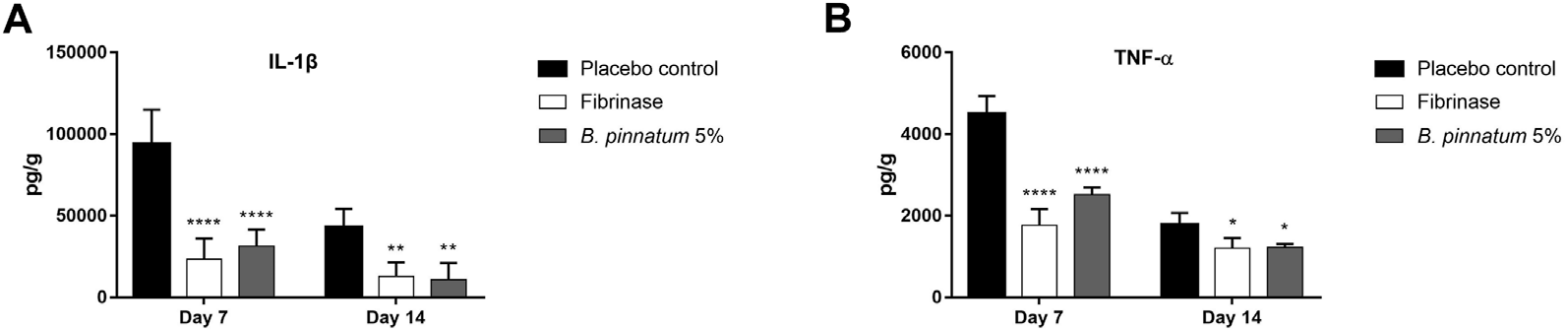
Effect of topical treatment with a gel formulation containing 5% of the *B. pinnatum* extract on the levels of cytokines **(A)** IL-1β and **(B)** TNF-α in skin wound biopsies. Results expressed as mean ± standard error of mean (*n* = 5/group). ****p < 0.0001, **p < 0.01, and *p < 0.05 *vs*. placebo control by one-way ANOVA (Tukey’s post-test).

Figure 5A and Figure 5B show an increase in the levels of the pro-inflammatory cytokines IL-1β and TNF-α in the biopsies of the skin wound collected on the 7^th^ and 14^th^ day of the control group treated with placebo gel. Topical treatment with a gel formulation containing *B. pinnatum* leaf extract was able to significantly reduce the levels of these cytokines on the 7^th^ (IL-1β, *p* < 0.0001 and TNF-α, *p* < 0.0001) and 14th (IL-1β, *p* < 0.01 and TNF-α, *p* < 0.05) days compared to the placebo gel control group, consequently decreasing the local inflammatory process. As expected, treatment with Fibrinase^®^ also showed a statistically significant result in the reduction of the levels of these cytokines on the 7th (IL-1β, *p* < 0.0001 and TNF-α, *p* < 0.0001) and 14th (IL-1β, *p* < 0.01 and TNF-α, *p* < 0.05) days compared to the placebo control group.

#### 3.4.3 Histological analysis of skin wound healing in rats

**FIGURE 6:**
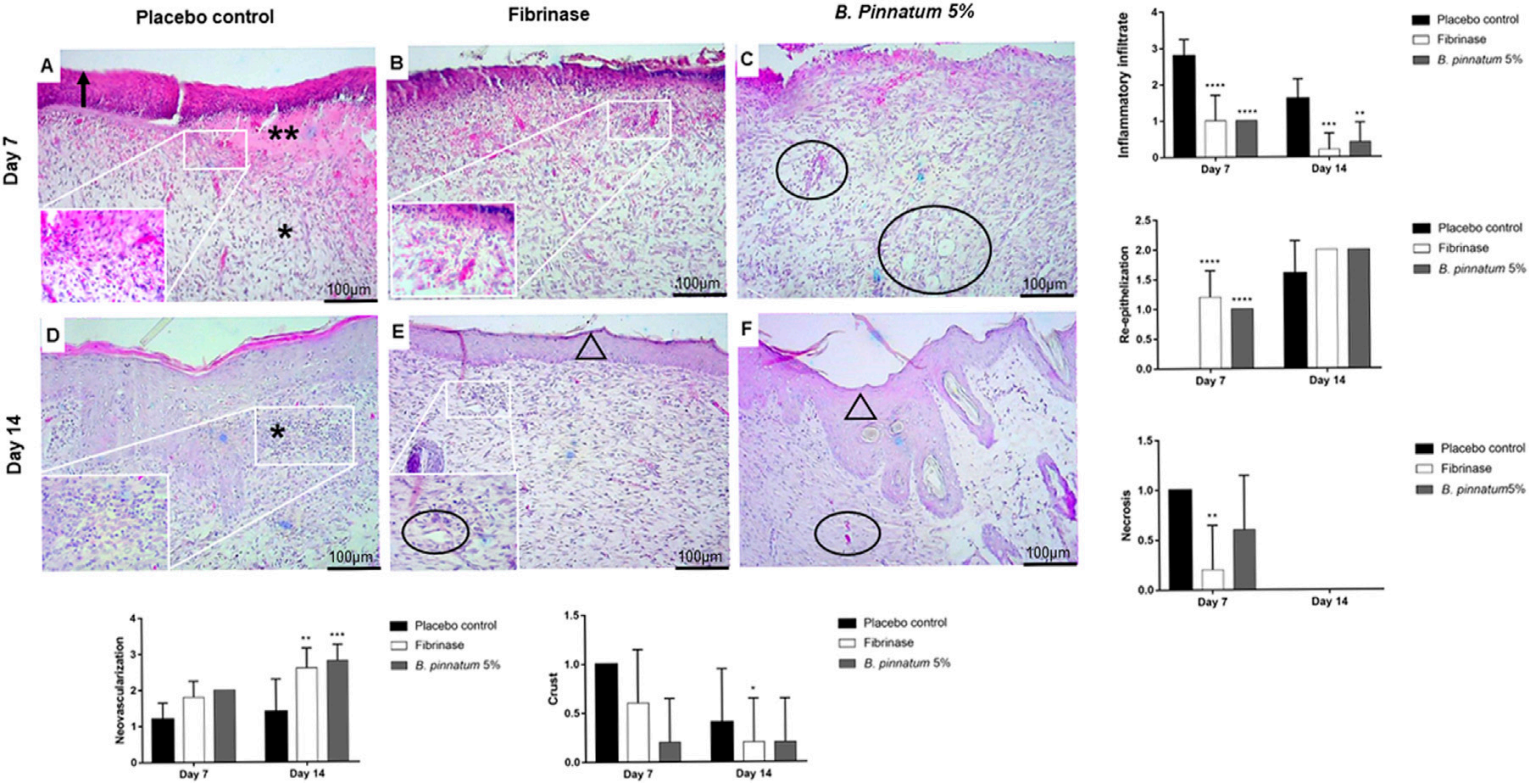
Histological characteristics of representative samples of skin wound biopsies from the back of Wistar rats. Hematoxylin–eosin (H&E) staining showing the sectioning of the biopsies in the transverse direction. View under a brightfield microscope at 10 × magnification and zoom at 40 × magnification. Placebo gel, Fibrinase^®^, and gel containing *B. pinnatum* leaf extract groups evaluated on day 7 [**(A,B,C)** respectively] and on day 14 [**(D,E,F,)** respectively]. Arrow indicates crust. Two asterisks indicate fibrin and coagulation region. An asterisk indicates inflammatory infiltrate. Circle indicates neovascularization. Triangle indicates re-epithelialization. Statistical difference demonstrated by Mann–Whitney test (*n* = 5), *p < 0.05, **p < 0.01, ***p < 0.001, and ****p < 0.0001 *vs*. placebo control.

Histological analysis of the wound tissue from the placebo control group showed intense inflammatory infiltrate and a purulent fibrin exudate characteristic of the inflammatory phase of the healing process, and this is possible to visualize in the analysis of the samples collected on the 7th day (Figure 6A). In the histopathological analysis of the group treated topically with the gel formulation containing 5% of the *B. pinnatum* extract, focal areas of inflammatory infiltrate were observed besides the re-epithelialization area and formation of new vessels, characteristic of the proliferative phase of the healing process (7th and 14th days, Figures 6C, F, respectively). The group treated with Fibrinase^®^ also showed focal areas of inflammatory infiltrate besides the re-epithelization and formation of new vessels (7th and 14th days, Figures 6B, E, respectively). On the 14th day, it was still possible to observe a slight inflammatory infiltrate in the placebo control group (Figure 6D), while the remodeling phase was more advanced in the groups treated with the gel formulation containing *B. pinnatum* leaf extract and Fibrinase^®^ (Figures 6E, F, respectively).

#### 3.4.4 Effect of topical treatment with gel formulation containing *Bryophyllum pinnatum* leaf extract at 5% on vascular endothelial growth factor expression

**FIGURE 7:**
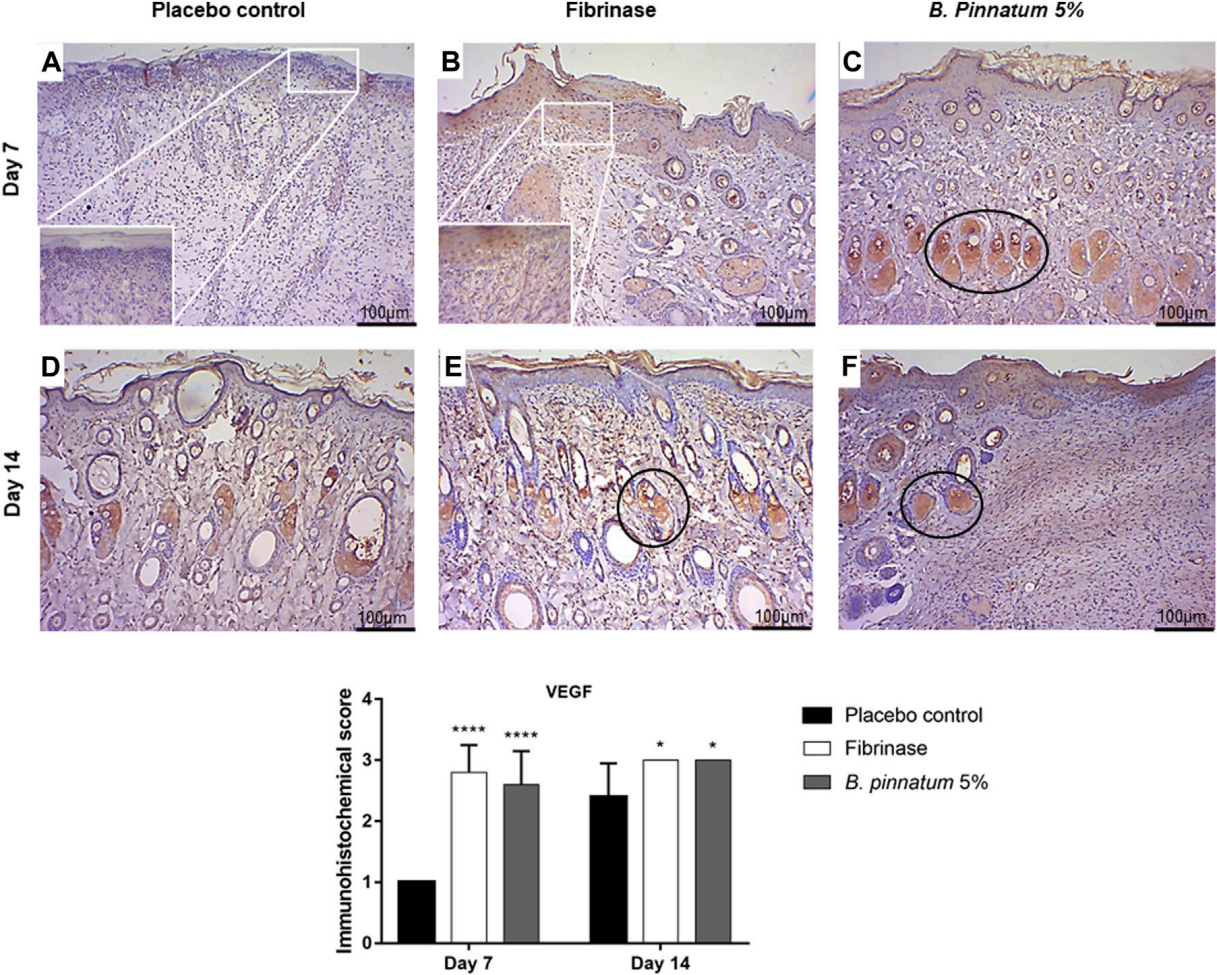
Immunohistochemical analysis of VEGF in skin biopsies showing the cut in the transverse direction. View under a brightfield microscope at 10 × magnification and zoom at 40 × magnification. Placebo, Fibrinase^®^, and gel containing *B. pinnatum* leaf extract at 5% control groups evaluated on day 7 **(A,B,C,** respectively) and on day 14 **(D,E,F,** respectively). Circle, strong expression of VEGF. Statistical difference demonstrated by Mann–Whitney test (*n* = 5), *p < 0.05 and ****p < 0.0001 *vs*. placebo control.

Immunohistochemical analysis for the VEGF marker did not reveal staining in the placebo control group treated with placebo gel in the samples collected on the 7th day (Figure 7A), and immunostaining with stronger brown staining was observed only in the samples collected on the 14th day (Figure 7D). When analyzing the figures that represent the topical treatment with the gel formulation containing *B. pinnatum* leaf extract, stronger immunostaining with a brown color was observed on the 7th and 14th days (Figures 7C, F, respectively). This indicated that the treatment topic with gel could stimulate the expression of the VEGF marker, a result that corroborates the histological analysis, where it is possible to observe the development of new vessels on the 7th and 14th days (Figures 6C, F, respectively). As expected, topical treatment with Fibrinase^®^ was also able to increase the expression of the VEGF marker on the 7th and 14th days (Figures 7B, E, respectively). This also corroborates with results found in the histopathological analysis, in which it is possible to observe the development of new vessels on the 7th and 14th days (Figures 6B, E, respectively).

## 4 Discussion

In this study, an essential quality control parameter was established, the total phenolic and flavonoid content per gram of a wound healing formulation in gel containing *B. pinnatum* leaf extract. The association of TPC and TFC per gram of gel formulation is a required parameter to ensure the safety, efficacy, quality, and batch-to-batch reproducibility of the final products. Few data about the quantification of markers in a formulation containing botanical extracts (Zainol et al., 2021) have been reported in the literature.

In addition, the data provide preliminary evidence for the stability of a formulation in gel containing *B. pinnatum* extract according to physical parameters pH, water activity, and phytochemical and biological stability at least during the storage period of 30 days at room temperature (on the bench) and at a controlled temperature (40°C) and relative humidity (75%). A few chemical content changes were observed at accelerated conditions but kept a similar chromatographic chemical profile compared to free extract. Besides that, in the biological stability analysis, the solutions extracted from gel containing *B. pinnatum* leaf extract could inhibit ROS production in the same way as the *B. pinnatum* leaf extract. In the literature, it is described that phenolic compounds inhibit the inflammatory process by decreasing ROS production and, consequently, the synthesis and release of pro-inflammatory cytokines (Esposito et al., 2014). Further long-term stability studies are required to prove phytochemical stability and/or improve the formulation in gel to avoid microbiological growth.

In the literature, it is described that the main phenolic compounds identified in the leaves of the species *B. pinnatum* are the glycosylated flavonoids derived from quercetin, kaempferol, and luteolin (Muzitano, 2006; Cruz et al., 2008; El Abdellaoui et al., 2010; Tatsimo et al., 2012; Darmawan, 2013; Aoki et al., 2014; Chibli et al., 2014; Fernandes et al., 2016; De Araújo et al., 2018; Dos Santos Nasimento et al., 2018; Fernandes et al., 2019; Andrade et al., 2020; De Araújo et al., 2021). Furthermore, the content of three flavonoids of *B. pinnatum* aqueous leaf extract, quercetin 3-*O*-*α*-L-arabinopyranosyl-(1→2)-*O*-*α*-L-rhamnopyranoside (Bp1), kaempferol 3-*O*-*α*-L-arabinopyranoside (1→2)-*O*-*α*-L-rhamnopyranoside (Bp2), and quercetin 3-*O*-*α*-L-rhamnopyranoside (Bp3) was previously determined, in which Bp1 was the major compound identified (De Araújo et al., 2021). According to previous studies (Dos Santos Nascimento et al., 2018; Coutinho et al., 2021), the results of this analysis confirmed that Bp1 is considered an analytical marker of this species. It is important to mention that Bp1 has shown anti-inflammatory (Lourenço et al., 2020), wound healing (Coutinho et al., 2021), gastroprotective effect (De Araújo et al., 2018), and leishmanicidal activities (Muzitano et al., 2006; Ferreira et al., 2014). In addition, Bp1 was not identified in *Kalanchoe laciniata* (syn: *Kalanchoe brasiliensis*) leaf extract (Fernandes et al., 2016), a similar morphological species to *B. pinnatum*. In popular use, both species are used as if they were the same plant. Then, for quality control, Bp1 is a good selective marker to differentiate these species. Another important issue is its high content in the extract compared to other flavonoids. This chemical marker is also a good choice to apply in the supply chain, from cultivation to the final product.

Phenolic compounds have attracted research attention by being one of the principal components in botanical extracts responsible for their anti-inflammatory capacity (Ambriéz-Prez et al., 2016). Among phenolic compounds, flavonoids are found in different plant species (Glevitzky et al., 2019) and can act as an antioxidant through a free radical scavenging mechanism (Dangles et al., 2000). In the literature, it is also described that flavonoids have pharmacological activities related to the inhibition of the proliferation of inflammatory cells and, consequently, the suppression of the expression of pro-inflammatory cytokines, in addition to decreasing the excessive production of ROS by reducing the concentration of nitric oxide and stimulating the activity of endogenous oxidative enzymes (Antonisamy et al., 2016; Wang et al., 2017). In this sense, *B. pinnatum* leaf is a good source of flavonoids; it is a small plant and easy to establish cultivation on a large scale. Its traditional use for treating inflammatory diseases highlights the need to follow up on in-depth research with this plant.

Our findings provide strong evidence that topical treatment with a gel formulation containing *B. pinnatum* leaf extract at 5% was able to reduce the wound area, also decreasing the levels of the pro-inflammatory cytokines TNF-α and IL-1β. These findings suggest that the *B. pinnatum* formulation stimulated the healing process and decreased excessive inflammation at the injured site. As expected, these findings were also observed with the topical treatment of Fibrinase®. According to Kondo and Ishida (2010), TNF-α and IL-1β are pro-inflammatory cytokines that play an important role in the initial phase of the wound healing process (Kondo and Ishida, 2010). TNF-α plays an important role in reducing the development of tissue granulation and in the organization of collagen fibers. However, for efficient healing, it is desirable to increase the production of granulation tissue rich in cells and vessels for the remodeling phase to take place (Kondo and Ishida, 2010; Gragnani et al., 2013). In this context, the immunomodulation of these cytokines observed with topical treatment using the gel formulation containing *B. pinnatum* extract may have contributed to the increase in granulation tissue formation and consequently accelerated wound closure. Considering that *B. pinnatum* is rich in flavonoids, it is possible to hypothesize that these compounds are responsable at least in part for their healing properties. According to Ueda et al. (2004), several theories are described in the literature, reporting the relationship between the anti-inflammatory potential of flavonoids and their structures. All these theories were built on the basis of hydroxyl groups, especially on the A and B rings. The presence of hydroxyl groups on C-5 and C-7 on the A-ring and C-4 on the B-ring is associated with the inhibition of the synthesis of TNF-α.

In addition to the parameters mentioned previously, topical treatment with a gel formulation containing *B. pinnatum* extract was also able to reduce the inflammatory infiltrate on the 7th day of treatment and stimulated the development of new vessels in the histological analysis with hematoxylin and eosin. This last finding corroborates the immunohistochemical analysis, in which the increase in VEGF expression was verified. According to Ferrara et al. (2003) and Staels et al. (2019), VEGF belongs to a family of homodimeric proteins that consists of at least six members: VEGF-A (VEGF), VEGF-B, VEGF-C, VEGF-D, VEGF-E, and placental growth factor (PIGF). VEGF is the most abundant form and plays important roles in endothelial cell proliferation, migration, and activation, as well as promoting permeability, collagen synthesis, and angiogenesis (Ferrara et al., 2003; Staels et al., 2019). Therefore, when analyzing all the parameters observed in the evaluation of the healing activity of wounds in this study, it is possible to suggest that *B. pinnatum* has healing and anti-inflammatory potential in skin wounds.

It is important to mention that in this study, female rats were chosen because, according to Machowska et al. (2004), Chua et al. (2019), and McKay et al. (2022), sex hormones may affect the wound healing process in rodents. In these studies, it is described that testosterone appears to stimulate a prolonged inflammatory effect on wounds and cause a delay in healing. This delay in the healing process can cause a longer time of suffering to the animals; therefore, male animals may have a longer suffering time than females (Machowska et al., 2004; Chua et al., 2019; McKay et al., 2022). Based on preclinical evidence and the principles of animal ethics, we choose females to avoid this exacerbated time of suffering.

The findings obtained in this study corroborate with those of previous studies that also evaluated this species and observed immunomodulatory activity *in vitro* (Cruz et al., 2008; Cruz et al., 2012), phospholipase *in vitro* (Fernandes et al., 2016), anti-inflammatory *in vivo* (Ojewole, 2005; Cruz et al., 2008; Gupta et al., 2009; Afzal et al., 2012; Cruz et al., 2012; Chibli et al., 2014; Ferreira et al., 2014; De Araújo et al., 2019), and wound healing *in vivo* (Khan et al., 2004; Nayak et al., 2010; Lebedeva et al., 2017; Zakharchenko et al., 2017; Coutinho et al., 2021). It is also worth mentioning that one of the healing activities conducted previously used extraction with petroleum ether, ethanol, and water and treatment orally (Khan et al., 2004). The second used ethanolic extract and topical treatment using the extract, with Vaseline as a base (Nayak et al., 2010). The third used an aqueous extract of wild and transgenic *B. pinnatum* containing an *Agrobacterium tumefaciens* vector, topical application in wounds contaminated with *S. aureus* and *P. aeruginosa*, but not incorporated in a formulation and focusing on antimicrobial activity (Lebedeva et al., 2017). The fourth study used the aqueous extract of transgenic *B. pinnatum* containing a binary vector of *Agrobacterium tumefaciens* topical application in wounds contaminated with *C. albicans* and focused on antifungal activity, but the extract was not incorporated into a formulation (Zakharchenko et al., 2017). Finally, Coutinho et al. (2021) showed a good healing effect of two creams formulated, one containing *B. pinnatum* extract and another the Bp1 compound, through macroscopic and histological analysis. Thus, our study joins previous works to increase the knowledge about the healing effect of *B. pinnatum* extract in a formulation in gel and elucidate the understanding of its mechanism of action and phytochemical and biological stability.

Currently, the pharmacological potential of phenolic compounds and phenolic-rich extracts focused on the inhibition of chronic inflammatory diseases has excelled due their remarkable properties to act in multi-targets of the inflammatory process. Phenolic compounds are found in various foods and medicinal plants. Therefore, phenolic-rich botanical extracts can be a good therapeutic strategy to treat inflammation with minimal or no adverse side effects or in combination with approved drugs (Ambriz-Pérez et al. 2016; Shahidi and Yeo, 2018; De Moraes et al., 2020).

According to the results obtained in this study, the gel formulation containing *B. pinnatum* extract presented a total phenol content with a mean equal to 2.77 ± 0.06 mg (mg of gallic acid per 1 g of gel formulation) and total flavonoids with a mean equal to 1.58 ± 0.03 mg (mg of quercetin in 1 g per the gel formulation). Furthermore, the data provide convincing evidence that the major compound from *B. pinnatum* leaf extract, quercetin 3-*O*-*α*-L-arabinopyranosyl-(1→2)-*O*-*α*-L-rhamnopyranoside (Bp1) through a preliminary stability study, is a good marker choice in a final product due its stability and high content. In short, in the present work, two quality control parameters required by current herbal medicine regulations from Brazil (Brasil, 2014) were determined, the phytochemical content of products containing active botanical extracts and some parameter of stability acceptance criteria.

To support this work, the development, manufacturing, and quality control of formulations containing botanical extracts with potential therapeutic effects are of great importance for guaranteeing the quality, safety, and efficacy of the finished product (Mukherjee et al., 2015; Klein-Júnior et al., 2021). In this perspective, our results about a wound healing gel formulation containing *B. pinnatum* aqueous leaf extract can support further studies to develop a novel topical wound healing product containing active *B. pinnatum* leaf extract in the pharmaceutical field.

## 5 Conclusion

Topical treatment with a gel formulation containing *B. pinnatum* aqueous leaf extract at 5% accelerated the healing process of skin wounds induced on the back of rats. In this trial, the reduction in wound area was accompanied by a decrease in inflammatory infiltrate, besides a reduction in the levels of the pro-inflammatory cytokines IL-1β and TNF-α. The development of new vessels by histological analysis and increased expression of the VEGF marker by immunohistochemistry was also observed, indicating that topical treatment with *B. pinnatum* stimulates wound healing.

The formulation in gel showed phytochemical and biological stability for 30 days and proved that the main compound of *B. pinnatum* leaf extract, quercetin 3-*O*-α-L-arabinopyranosyl-(1→2)-*O*-α-L-rhamnopyranoside is a good chemical marker to be applied in the quality control of botanical extracts and products containing this species.

Therefore, our results reinforce the healing potential of this plant to be applied as a novel active pharmaceutical ingredient. However, it is necessary to conduct clinical studies to evaluate safety and efficacy in humans.

## Data Availability

The raw data supporting the conclusion of this article will be made available by the authors, without undue reservation.
